# Prevalence and characterization of verotoxigenic-*Escherichia coli* isolates from pigs in Malaysia

**DOI:** 10.1186/1746-6148-9-109

**Published:** 2013-06-04

**Authors:** Wing Sze Ho, Lai Kuan Tan, Peck Toung Ooi, Chew Chieng Yeo, Kwai Lin Thong

**Affiliations:** 1 Institute of Biological Sciences, Faculty of Science, University of Malaya, 50603, Kuala Lumpur, Malaysia; 2 Laboratory of Biomedical Science and Molecular Microbiology UMBIO Research Cluster, University of Malaya, 50603, Kuala Lumpur, Malaysia; 3 Department of Clinical Veterinary Studies, Faculty of Veterinary Medicine, University Putra Malaysia, 43400, Serdang, Selangor Darul Ehsan, Malaysia; 4 Faculty of Agriculture and Biotechnology, Universiti Sultan Zainal Abidin, 21300, Kuala Terengganu, Malaysia

**Keywords:** Verotoxigenic-*E. coli* (VTEC), Shiga-toxin (Stx)-producing *E. coli* (STEC), Postweaning diarrhea (PWD), Multidrug resistance, Pig farms, Swine

## Abstract

**Background:**

Postweaning diarrhea caused by pathogenic *Escherichia coli*, in particular verotoxigenic *E. coli* (VTEC), has caused significant economic losses in the pig farming industry worldwide. However, there is limited information on VTEC in Malaysia. The objective of this study was to characterize pathogenic *E. coli* isolated from post-weaning piglets and growers with respect to their antibiograms, carriage of extended-spectrum beta-lactamases, pathotypes, production of hemolysins and fimbrial adhesins, serotypes, and genotypes.

**Results:**

PCR detection of virulence factors associated with different *E. coli* pathotypes (ETEC, EPEC, EHEC, and VTEC) revealed that VTEC was the only pathotype identified from six swine farms located at north-western Peninsular Malaysia. A low prevalence rate of VTEC was found among the swine samples (n = 7/345) and all 7 VTEC isolates were multidrug resistant. Five of these isolates from different hosts raised in the same pen were likely to be of the same clone as they shared identical sero-pathotypes (O139:H1, VT2e/α-*hly*/F18), resistance profiles and DNA fingerprinting profiles. Two other serotypes, O130: H26 (n = 1) and O168: H21 (n = 1) carrying virulence factors were also identified. O168: H21 is possibly a new serotype as this has not been previously reported.

**Conclusions:**

The occurrence of VTEC with infrequently encountered serotypes that are multidrug resistant and harbouring virulence factors may be of public health concern. The detection of possible clones in this study also showed that the combination of different typing tools including phenotyping and genotyping methods is useful for molecular epidemiologic surveillance and studies.

## Background

*Escherichia coli* is the leading cause of morbidity and mortality in newborn and weaned pigs [1] with postweaning diarrhea (PWD) as the main health problem in piggeries and has caused significant losses in the swine industry worldwide [2]. The main causal agents of PWD are verotoxin (VT)-producing *E. coli* (VTEC), also known as Shiga-toxin (Stx)-producing *E. coli* (STEC) and enterotoxigenic *E. coli* (ETEC) [2]. Enterohemorrhagic *E. coli* (EHEC) and enteropathogenic *E. coli* (EPEC) have also been found associated with PWD [3]. VTEC is characterized by the production of VTs (VT1 and VT2) which disrupt protein synthesis whereas ETEC is characterized by the production of heat labile enterotoxin (LT) and heat stable enterotoxin (ST) [4]. Among the pathogenic *E. coli*, VTEC O157 is often the main focus of most surveillance programs due to its association with severe human infections. However, monitoring should be extended to other serotypes as cases of human infections caused by non-O157 VTEC are substantial [5]. Furthermore, serotypes involved in porcine PWD were mostly non-related to human infections [6,7].

Pathogenicity of *E. coli* is associated with bacterial fimbrial adhesins which enable the bacteria to adhere to the cell lining of the small intestine and colonize the host [4]. A number of fimbrial adhesins have been associated with PWD caused by pathogenic *E. coli* in pigs namely the F4, F5, F6, F17, F18 and F41 fimbriae [8,9] with F4 and F18 as the most common adhesins [2,9,10]. Despite the substantial number of swine farms in Malaysia, there is a dearth of published information on the characteristics of pathogenic *E. coli* infection in pigs from Malaysian farms. Hence, the objectives of this study were to determine the prevalence of different *E. coli* pathotypes in Malaysian swine samples and to further characterize the pathogenic *E. coli* with respect to their antibiograms, production of extended spectrum β-lactamases (ESBLs), virulence genes, fimbrial adhesive genes, serotypes, production of hemolysins and genetic diversity. The outcome of this study would provide information on the prevalence and characteristics of pathogenic *E. coli* as a cause of post-weaning diseases in pigs in Malaysia.

## Results

### Isolation and confirmation of *E. coli* isolates from post-weaning piglets and growers

A total of 86 post-weaning piglets (< 2 months) and 24 growers (2–4 months) [from farms D (n = 20), E (n = 20), and F (n = 18) from the state of Perak and farms G (n = 16), H (n = 20), and I (n = 16) from Penang were selected and three swab types (nasal, tongue and rectal swabs) were sampled from each pig. A total of 511 presumptive *E. coli* isolates were obtained from these 110 pigs. Out of 511 presumptive *E. coli* isolates, 345 (67.5%) were confirmed as *E. coli* as they were *phoA*-positive by PCR [11] (nasal swabs, n = 57; rectal swabs, n = 202 and tongue swabs, n = 86).

### Virulence and adhesion genes

**Figure 1:**
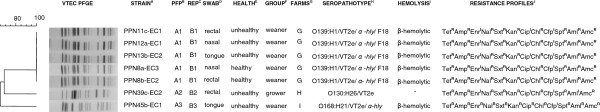
**Dendrogram generated from PFGE profiles of the VTEC strains using UPGMA based on Dice coefficients of similarity.**^A^Strain code; ^B^pulsotype; ^C^REP profiles; ^D^swab samples; ^E^health condition of pig; ^F^age group; ^G^swine farm; ^H^serotype/virulence factor/hemolysin gene/adhesin; ^I^phenotypic result for haemolytic activity on washed blood agar; ^J^antimicrobial resistance profiles.

None of the 345 isolates was positive for the LT1, LT2, ST and *eaeA* genes while 9 isolates were verotoxin (VT)-positive and were thus termed as verotoxin-producing *E. coli* (VTEC). Of the 9 VTEC isolates, 4 were obtained from the same swab samples of 2 animals (i.e., duplicates), hence, only one VTEC isolate from each animal was further characterized. Overall, 7 VTEC isolates were subjected to further characterization. These 7 VTEC isolates were obtained from three swine farms located in Penang and no VTEC was found in the farms that were sampled in Perak. Of the 7 VTEC, 5 were obtained from farm G (located in the coastal area) and 1 each from farms H and I (located inland). VTEC were obtained from all three swab types (rectal: n = 3; tongue: n = 2; nasal: n = 2) (Figure 1). Five VTEC were isolated from unhealthy pigs (from 5 different hosts) while 2 were from a healthy pig (2 different swab samples from a single host) (Figure 1). All the VTs belonged to subtype 2e (VT2e). Only 1 type of fimbrial gene, F18 was detected in 5 of the 7 VTEC isolates (Figure 1) and none was positive for F4, F5, F6, F17 and F41.

### Serotyping of VTEC

**Figure 2:**
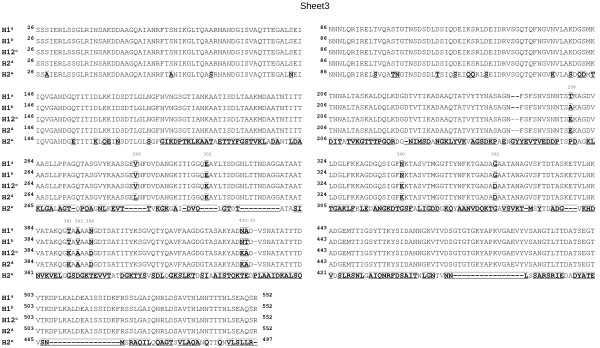
**Multiple-sequence alignment of flagellin amino acid sequences representing serogroups H1, H2 and H12 of *E. coli *using Clustal W.** The amino acid sequence of H1^a^ was obtained in this study [GenBank: KC753181]. The amino acid sequences of serogroups H1^b^, H12^c^,H2^d^ and H2^e^ were retrieved from NCBI protein database [GenBank: ADR27342, AAQ22679, AAD28525 and BAI30971, respectively]. Two very distinctive H2 flagellin sequences were included in the alignment *(H2*^*d*^*: common H2 sequence; H2*^*e*^*: peculiar H2 sequence obtained from E74/68).* A dash (‘-’) denotes a gap in the alignment. Amino acid sequence differences are indicated in bold letters. Numbers indicate the various amino acid sequence positions.

O antigen typing using both conventional and molecular methods revealed three O serogroups, namely O139, O130, and O168. The determination of the H serogroup for O139 *E. coli* isolates using molecular typing method was inconclusive as the amino acid sequence of the respective H antigens shared highest similarity with H1 (3 amino acid differences), H12 (7 amino acid differences) and a peculiar H2 flagellin (H2^d^ ,8 amino acid differences) (Figure 2). Very low degree of sequence identity was observed for the common H2 flagellin sequence (H2^e^) with the H antigen sequence obtained from our study (Figure 2). The respective RFLP of the *fliC* gene also resembled H1 as described previously by Fields *et al*., [12] (data not shown). However, the phenotypic result showed that the respective H antigen belonged to serogroup H2. Nevertheless, the H serogroup of these strains were designated H1 based on the molecular data since conventional H serotyping is reported to have discrepancy and uncertainty [13,14]. The representative *fliC* DNA sequence obtained in this study has been deposited in GenBank under the accession number KC753181.

Overall, H antigen typing using both conventional and molecular methods revealed the presence of serotypes O139:H1 (n = 5), O130:H26 (n = 1) and O168:H21 (n = 1) among the VTEC isolates. The most common serotype was O139:H1 which was isolated from different pigs in the same pen in farm G in Penang.

### Genotypes of VTEC based on PFGE and REP-PCR

Five out of 7 VTEC isolates shared an indistinguishable pulsotype designated A1 (Figure 1). These 5 VTEC isolates were obtained from the same farm and 2 of them were isolated from the same animal but from different swab samples. The remaining 2 isolates were isolated from 2 different farms in Penang and showed unrelated pulsotypes (more than 3 band differences). REP-PCR profiling also revealed only 3 distinct patterns with one REP profile found for each farm (Figure 1). Isolates that shared the same pulsotype (A1) had the same REP profile (B1). All 5 isolates with the same serotype shared an indistinguishable pulsotype and REP profile while another two were genetically different.

### Antibiograms and ESBL producers

All VTEC isolates were multidrug resistant as they were resistant to at least 3 groups of antimicrobials. They showed 100% resistance to Tet, Amp, Nal, Sxt, Kan, Chl, Spt, and Aml (Figure 1). However, none of the VTEC was ESBL positive.

### Haemolytic activity

Six out of the 7 VTEC isolates were beta-hemolytic as indicated by the zone of clearing on the defribinated, washed sheep blood agar after 4 hours of incubation. These 6 isolates were also positive for the α*-hly* gene. None of the isolates was positive for the *E-hly* gene.

## Discussion

ETEC and VTEC are common etiologic agents for PWD in pigs worldwide. However, the distribution of such pathogenic *E. coli* in the swine population in Malaysia is unknown and there is limited information for the Southeast Asian region. Different pathotypes of *E. coli* are known to be associated with specific virulence factors. Among the 5 virulence factors tested in this study, only verotoxin (VT) was detected and at a low prevalence of 2.0% (7/345). Thus VTEC appeared to be the only pathotype identified from the swine samples in north-western Peninsular Malaysia. However, a larger sample size would be needed in any future studies to estimate the true prevalence rates of pathogenic *E. coli* among the Malaysian swine populations. A recent study from Thailand [15] reported a relatively higher prevalence of VTEC (11.2%) from pigs with PWD. The results obtained in this study and that of Prapasarakul *et al.*[15] were in contrast with earlier reports by Zhang *et al.*[16] and Fairbrother *et al*. [2] who reported ETEC as the most common pathotype found in pigs with PWD in the United States and worldwide, respectively. The VT detected in this study belonged to subtype VT2e. Although Oanh *et al*. [17] reported that VT2e is commonly associated with oedema in pigs, however, none of the VTEC-infected pigs in this study showed signs of oedema. Nevertheless, pigs with oedema often suffer sudden death [17], hence these infected pigs are difficult to observe.

Of the 7 VTEC isolates, 6 were obtained from weaners and 1 from grower. Colonization of the guts of newly weaned piglets with pathogenic bacteria often occurs as the absorption and digestion of the gut is disturbed due to dietary changes [18]. A majority of the VTEC isolates (n = 5/7) were also isolated from unhealthy pigs inferring the undesirable outcome to the health conditions of the pigs due to infections.

H antigen typing for isolates with serogroup O139 yielded ambiguous result as the conventional serotyping method yielded H2, while the *fliC* sequence analysis closely matched to H1 type (with 3 amino acid differences). Serotyping has been reported to have limited sensitivity and specificity [13] and the result may not always be reliable. Hence, the H type for the O139 isolates was designated H1 (DebRoy, C., personal communication). The discrepancy in H serotyping results using both molecular and conventional serotyping methods had also been encountered by Reid *et al.*[14] for an EPEC strain (E74/68) with serotype O128:H2 which was investigated with respect to its *fliC* gene sequence. It was found that the amino acid sequence [GenBank: AAD28525] best resembles H1 (with 10 amino acid difference) and H12 (with 1 amino acid difference) out of the 527-amino acid residues of the flagellin protein (Figure 2) although the conventional serotyping revealed that it belongs to serogroup H2. However, the discrepancies in the result were not discussed by Reid *et al.*[14] in their paper. Coincidentally, the amino acid sequence of the “H2” flagellin obtained from strain E74/68 shared relatively high sequence identity with the H1 flagellin obtained in our study (8 amino acid difference) (Figure 2).

Swine VTEC has been reported to be associated with specific serogroups such as O138, O139 and O141 [19] and indeed, the major O serogroup identified in this study is O139. *E. coli* serotype O157:H7, which is commonly known to be a serious food-borne pathogen throughout the world, was not detected in this study. O139:H1, the most prevalent serotype in this study, has been reported worldwide to cause swine diseases such as PWD and oedema [2]. It has also been reported to be one of the major serotypes associated with diseased pigs [20]. The other two serotypes identified in this study were O130:H26 and O168:H21. O130:H26 is considered as a rare serotype as it has only been reported once and was isolated from the stool sample of a diarrhea patient [21] while *E. coli* with serotype O168:H21 has not been described in any study before. To the best of our knowledge, serotypes O130:H26 and O168:H21 have not been previously associated with swine diseases and are also considered as infrequently encountered *E. coli* serotypes.

VTEC producing F4 and F18 fimbrial adhesins are one of the most common pathogens associated with diarrheas in pigs worldwide [2,16]. In our study, the F18 fimbriae was the most common, which concurred with the findings of Vu Khac *et al*. [9] in Slovakia while other studies by Zhang *et al*. [16] and Madoroba *et al*. [22] showed that F4 was the prevalent type of fimbriae in pathogenic *E. coli* isolated from young piglets in the United States and Zimbabwe, respectively. The distribution of fimbriae types may vary for different geographical regions. Fairbrother *et al*. [2] also reported that ETEC serogroup O139 is often associated with the F18 fimbriae and these strains typically caused PWD in Australia and oedema in Europe. The detection of hemolysins of VTEC was also evaluated using molecular and phenotypic methods revealing the presence of α*-hly* but not *E-hly*. The presence of α*-hly* may serve as a virulence factor for our local VTEC isolates as it codes for pore forming cytolysins [2], which are capable of destroying red blood cells [23].

The emergence of multi-drug resistant bacteria in the poultry farming industry, including the piggeries is of public health concern as transmission of these bacteria to humans via the food-chain and direct contact has been described [24]. However, the prophylactic use of antimicrobials was justified by a study which demonstrated that the withdrawal of animal growth-promoting antimicrobials from feeds was associated with elevated prevalence of diarrhea, weight loss and increased fatality rate of post-weaning pigs mainly due to *E. coli* infections [25]. In this study, all VTEC isolated were multidrug-resistant, in which high resistance rates were observed for Amp, Aml, Cip, Enr, Tet, Sxt, Chl and Nal (Figure 1). This is similar to recent reports from Thailand and Korea [15,26]. This suggests that the swine farms in those countries may have used the similar antimicrobials for prophylactics as the swine farms in Malaysia.

PFGE, which is known for its discriminatory power as a molecular typing tool in epidemiologic studies revealed that 5 out of the 7 VTEC isolates shared an indistinguishable pulsotype, which was further supported by REP-PCR. In addition, these 5 VTEC isolates also shared identical seropathotypes, i.e., O139:H1 VT2e/ α*-hly*/ F18 and antimicrobial resistance profiles (Figure 1). All five isolates were obtained from different pigs raised in the same pen, strongly suggesting that the transmission of the *E. coli* clone among the animals have occurred, and this transmission may occur via direct contact (such as nose-to-nose), contaminated aerosols [27] or other mechanisms.

## Conclusions

In conclusion, VTEC was the only pathogenic *E. coli* identified among the pigs sampled in this study. A clone with serotype O139:H1 that is commonly associated with diseased pigs worldwide was found in Malaysia. Two infrequently encountered VTEC serotypes, O130:H26 and O168:H21 carrying virulence factors were also identified. The VTEC isolates were all multidrug resistant and a majority possessed the fimbrial adhesins, F18 and hemolysins which may serve as additional virulence factors. More samples should be characterized in any future study to determine the true prevalence of different pathogenic *E. coli* obtained from Malaysian swine populations.

## Methods

### Sampling sites and isolation of bacterial isolates

Random selection of participating swine farms was not possible in this routine observational study as selection was subjected to management and practices of the commercial swine farms. A total of six swine farms located in two states in the north-western part of Peninsular Malaysia, i.e., Perak (farms D, E, F) and Penang (farms G, H, I) were enrolled in this study during year 2010 to 2011. Penang and Perak are the top two largest and important swine producing states in Malaysia and these farms are located in-land as well as by the coastal areas. We performed a stratified random sampling on two age categories of pigs (weaner, 1–2 months and grower, 2–4 months). Three types of swabs were sampled from each pig (nasal, tongue and rectal swabs) and maintained in Cary-Blair transport medium (Oxoid, UK) in ice box before being processed in laboratory. Presumptive *E. coli* were determined by direct streaking on Chromagar *E. coli* selective medium, CHROMagar™ ECC (CHROMagar™, Paris, France) which give the characteristic blue-colored morphology (according to manufacturer)*.* One to 3 presumptive *E. coli* colonies on CHROMagar™ ECC were picked for further confirmation*.* Data of each pig included herd, age group, gender and clinical conditions (unhealthy/healthy) was recorded as observed by farmers and examined by a veterinarian. Pigs were termed unhealthy based on the common visible gastrointestinal clinical symptoms such as lethargy (standing with heads down), fever (>39.5°C), diarrhea, rectal prolapse, blood from rectum and dehydration. Healthy pigs are pigs without any of the above-mentioned gastrointestinal clinical symptoms. Disabled pigs (eg: with conjunctivitis, crippled) were categorized as healthy pigs. All the samples were collected with supervision from institution veterinarian. The sampling complied with the current guidelines for the care and use of animals and was approved by the Animal Care and Use Committee (ACUC), Faculty of Veterinary Medicine, Universiti Putra Malaysia.

### PCR detection of housekeeping genes, virulence genes and adhesion genes of *E. coli* isolates

Boiled suspensions of presumptive *E. coli* cells were used as DNA template. All presumptive *E. coli* isolates (based on color morphology on CHROMagar™ ECC) were subjected to PCR confirmation targeting the housekeeping gene, *phoA* that is specific for *E. coli*[11]. For *E. coli* isolates that were positive for the *phoA* gene, another multiplex PCR to identify the virulence genes was performed. The virulence genes screened included the verotoxin (VT), heat-labile toxin 1 (LT1), heat-labile toxin 2 (LT2), heat stable toxin (ST) and attachment and effacement (*eaeA)* which are associated with the pathotypes VTEC, ETEC, EPEC and EHEC [11]. Three positive control strains (*E. coli* O157, SA53 and ATCC35401) were included in this study [11]. Any VT-positive *E. coli* isolates termed VTEC were further subtyped to VT1 or VT2 by using primers that were previously described [28] and subsequent DNA sequencing of the VT gene amplicon. PCR detection of the fimbrial colonization antigens (F4, F5, F6, F17, F18 and F41) was carried out on all VT-positive *E. coli* isolates as previously described [9,29,30].

### Serotyping

Both conventional and molecular serotyping were carried out for the VTEC isolates. Conventional serotyping using antisera was performed at Ipoh Public Health Laboratory (reference laboratory for *E. coli* serotyping in Malaysia) while molecular serotyping of O-serogroups and H-serogroups were determined by PCR-restriction fragment length polymorphism (RFLP) of the amplified O-antigen gene cluster (*rfb –* RFLP) [31] and flagellin-encoding (*fliC*) gene (*fliC -* RFLP) [12,32], respectively. Restriction digestion of the O-antigen and H-antigen amplicons was then performed using *Rsa*I (Promega, Madison, WI) (for H-antigen) and *Mbo*II (Promega, Madison, WI) (for O-antigen). The resultant RFLPs were then compared with the banding patterns described previously [31,32]. The H-antigen was further confirmed by performing DNA sequencing of the amplified *fliC* gene at a commercial facility (First BASE Laboratories).

### Genotyping by pulsed-field gel electrophoresis and repetitive extragenic palindrome (REP)-PCR

Pulsed-field gel electrophoresis (PFGE) for *Xba*I*-*digested genomic DNA of VTEC was performed in a CHEF Mapper (Bio- Rad, Hercules, CA) according to Thong *et al*. [33] using pulse times of 6.76 to 35.38 sec. *Xba*I*-*digested *Salmonella enterica* serovar Braenderup H9812 was used as the DNA size marker. PFGE was repeated twice to determine reproducibility. For untypeable isolates, 50 μM thiourea (Sigma Aldrich, USA) was added to the 0.5 × TBE buffer prior to PFGE run as described by Römling and Tümmler [34].

A dendrogram based on the pulsotypes was constructed with BioNumerics 6.0 (Applied Maths, Kortrijk, Belgium) using the Dice coefficient and unweighted pair group method using arithmetic averages (UPGMA) at 1.0% tolerance level. REP-PCR was carried out using REP oligonucleotides (Operon Biotechnologies GmBH, Germany) as previously reported [35].

### Antimicrobial susceptibility testing and phenotypic detection of ESBL for VTEC

Antimicrobial susceptibility testing (AST) of the VTEC isolates was performed using the disk diffusion method following the procedures described by the Clinical and Laboratory Standards Institute (CLSI) guidelines [36] on Mueller-Hinton II agar (BD) with commercial disks (Oxoid Ltd.). The antimicrobial agents used were: amikacin (Amk, 30 μg), amoxicillin/clavulanic acid (Amc, 20/10 μg), ampicillin (Amp, 10 μg), aztreonam (Atm, 30 μg), ceftriaxone (Cro, 30 μg), cefoperazone (Cfp, 30 μg), cefotaxime (Ctx, 30 μg), cefepime (Fep, 30 μg), ceftazidime (Caz, 30 μg), chloramphenicol (Chl, 30 μg), ciprofloxacin (Cip 5 μg), imipenem (Ipm, 10 μg), kanamycin (Kan, 30 μg), meropenem (Mem, 10 μg), nalidixic acid (Nal, 30 μg), tetracycline (Tet, 30 μg) and trimethoprim/sulfamethoxazole (Sxt, 30 μg). Four other commonly used antimicrobials in swine farming were also included: amoxicillin (Aml, 25 μg), colistin sulphate (Css, 10 μg ), enrofloxacin (Enr, 5 μg) and spectinomycin (Spt, 100 μg).

All VTEC isolates were also screened for ESBL-production using the modified double-disk synergy test (DDST) [37] and two E-test ESBL strips (CTX/CTX + clavulanic acid and CAZ/CAZ + clavulanic acid) (AB Biodisk). *E. coli* ATCC 25922 and ATCC 35218 were used as control strains in the ESBL phenotypic detection test.

### Phenotypic and genotypic characterization of hemolysins

Hemolytic activity of the VTEC isolates was determined by plating the cultures on trypticase soy agar (TSA) (Oxoid Ltd., Hampshire, England) supplemented with 5% defribinated sheep blood (Oxoid Ltd., Hampshire, England) where the blood was washed three times with saline. The inoculated plates were incubated for 4–6 hours (for the phenotypic detection of α-hemolysin) and 18–24 hours (for the phenotypic detection of enterohemolysin) at 37°C [38,39]. PCR detection of two hemolysin determinants [α*-hly* (codes for α-hemolysins) and *E-hly* (codes for enterohemolysin)] was also carried out as described [38,39].

## Competing interests

The authors declare that they have no competing interest.

## Authors’ contributions

WSH designed the study, analysed and interpreted data and wrote the manuscript. WSH and LKT performed research. WSH, LKT, PTO, CCY and KLT helped in drafting and critically reviewed the manuscript and contributed important intellectual output. PTO and KLT provided funding for the project. CCY and PTO co-supervised the project while KLT also designed, interpreted and supervised the project. All authors contributed to editing of the manuscript and all authors read and approved the final manuscript.
